# Sustained response to pembrolizumab without prior chemotherapy in high-grade serous ovarian carcinoma with *CSMD3* mutation

**DOI:** 10.1016/j.gore.2020.100600

**Published:** 2020-06-15

**Authors:** Julie Terzic, Amanda Seipel, Jean Dubuisson, Jean-Christophe Tille, Petros Tsantoulis, Alfredo Addeo, S.Intidhar Labidi-Galy

**Affiliations:** a Department of Oncology, Hôpitaux Universitaires de Genève, Genève, Switzerland; b Department of Diagnosis, Division of Clinical Pathology, Hôpitaux Universitaires de Genève, Genève, Switzerland; c Department of Woman, Child and Adolescent, Division of Gynecology, Hôpitaux Universitaires de Genève, Genève, Switzerland; d Department of Medicine, Faculty of Medicine, University of Geneva, Genève, Switzerland

**Keywords:** Pembrolizumab, PD1, High-grade serous ovarian carcinoma, CSMD3, Immunotherapy, CD8, CCL5, CXCL9

## Abstract

• Metastatic *CSMD3* mutated HGSOC showed objective and sustained response to pembrolizumab.
• The tumor was massively infiltrated by CD8^+^ T cells while PD-L1 TPS was at 10%.
• *CSMD3* mutated HGSOC showed up-regulation of *CCL5* and *CXCL9.*

## 1 Introduction

Epithelial ovarian cancer (EOC) is the leading cause of death among gynecologic malignancies (Siegel et al., 2018). High-grade serous ovarian carcinoma (HGSOC) is the most frequent subtype of EOC. It is mainly diagnosed at advanced stages (III/IV) and has poor outcome. Disease is usually revealed by non-specific abdominal symptoms related to peritoneal carcinomatosis such as abdominal pain, constipation and ascitis. Pleural metastases are observed in about 15% of HGSOC and median survival for these patients does not exceed 2 years (Hjerpe et al., 2018). Standard of care for patients with HGSOC comprises up-front cytoreductive surgery followed by platinum-taxane based chemotherapy. Top mutated genes in HGSOC according to the cancer genome atlas (TCGA) are *TP53*, *CSMD3* (CUB and Sushi multiple domains protein 3) and *BRCA1* (Cancer Genome Atlas Research, 2011).

Although immune checkpoint inhibitors directed against PD1 and PD-L1 have been a breakthrough therapy in several cancers, this has not been the case for EOC, with an objective response rate (ORR) lower than 10% (Matulonis et al., 2019). Here, we report the case of a woman with FIGO stage IVA HGSOC (pleural metastases) and *CSMD3* mutation, initially misdiagnosed as metastatic lung carcinoma, who showed prolonged response to monotherapy with anti-PD1 pembrolizumab.

## 2 Case

A 75-year-old female, never smoker, with a medical history of end-stage kidney failure (creatinine clearance at 12 ml/min), diverticulitis and hypertension related to hereditary polycystic kidney and liver disease, presented to the Emergency Room at the University Hospital of Geneva in August 2017 with abdominal pain located in the lower left quadrant. Computed tomography (CT-scan) of the chest and abdomen without contrast medium injection, due to renal failure, revealed an acute sigmoid and rectal diverticulitis. The diverticulitis was treated with ceftriaxone and metronidazole IV and responded favorably.

**Fig. 1:**
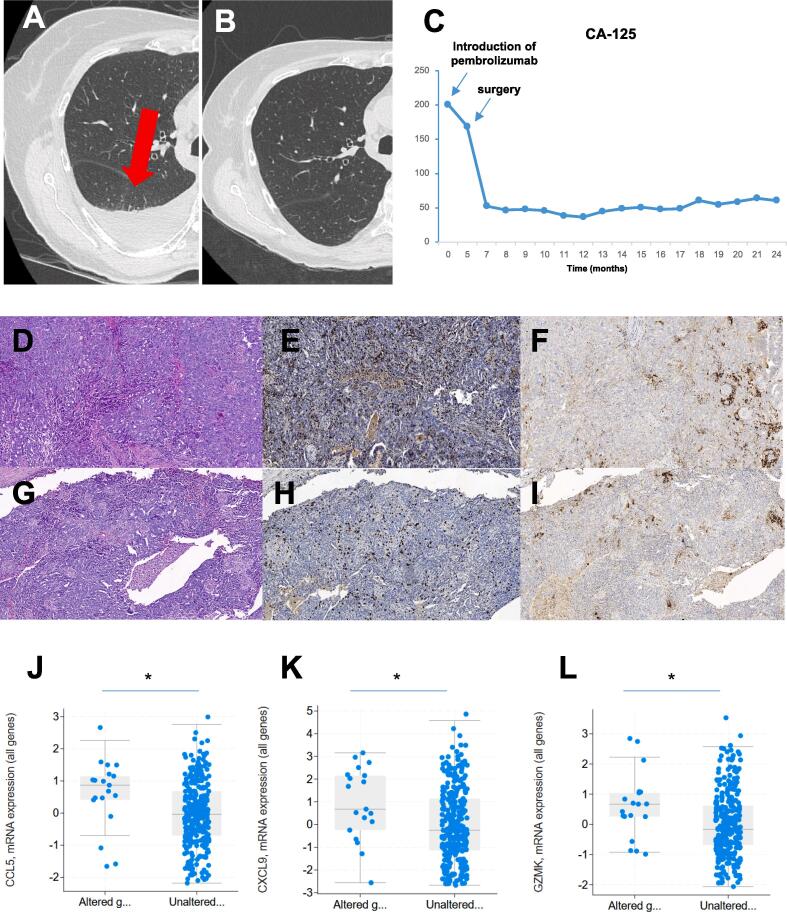
Prolonged response of *CSMD3* mutated metastatic HGSOC to pembrolizumab. A-B. Chest CT-scan revealed pleural effusion that regressed after 2 months of treatment with pembrolizumab. C. CA-125 trend on therapy. D-F. H&E, CD8 and PD-L1 staining of the first biopsy (pleural metastasis) diagnosed as a poorly differentiated adenocarcinoma of probable pulmonary origin, with massive infiltration by CD8^+^ lymphocytes and focal PD-L1 positivity (X20). G-I. H&E, CD8 and PD-L1 staining of the ovarian tumor (HGSOC) after 5 months of treatment with pembrolizumab, showing overlapping morphology, massive infiltration by CD8+ lymphocytes and focal PD-L1 positivity (X20). J-L. Boxplots of *CCL5*, *CXCL9* and *GZMK* mRNA levels in *CSMD3* mutated (altered) and non-mutated (unaltered) HGSOC in the TCGA cohort. *: *p* < 0.05.

Although the patient had no respiratory symptoms, the chest CT-scan revealed a right pleural effusion associated with multiple pleural nodules (Fig. 1A). Thoracentesis showed exudative fluid but no malignant cells. She underwent thoracoscopy with biopsies of pleural lesions. Histopathological analyses revealed a poorly differentiated adenocarcinoma. Immunohistochemistry (IHC) showed positive staining for pancytokeratin, cytokeratin 7, EMA, ALK and BerEP4. Cancer cells did not express cytokeratin 20, TTF1, GATA-3, vimentin, CDX-2, calretinin or CD10. Further molecular study by FISH did not show *ALK* translocation. The PD-L1 Tumor Proportion Score (TPS) was estimated to 10%. The diagnosis of a primary lung adenocarcinoma metastatic to the pleura was retained.

Between December 2017 and March 2018, Alectinib, a pan-ALK inhibitor was initiated, based on the ALK expression by IHC and the ALex trial (Peters et al., 2017). A follow-up CT-scan showed progression of the pleural effusion despite stability of pleural nodules leading to suspension of Alectinib. In April 2018, the patient presented again to the Emergency Room with a new episode of acute abdominal pain and diarrhea. Abdominal CT-scan revealed an acute sigmoid diverticulitis complicated by infectious peritonitis and the presence of a suspect left ovarian mass, considered as ovarian metastasis of the lung adenocarcinoma. Paracentesis confirmed the diagnosis of infectious peritonitis but did not reveal malignant cells. The patient received 10 days of IV antibiotherapy (imipenem). The evolution was rapidly favorable with resolution of the symptoms. Pembrolizumab 200 mg IV every 3 weeks was initiated in May 2018 after clearing of diverticulitis symptoms. Twelve weeks after initiation of pembrolizumab, a new CT-scan showed that pleural nodules were stable but pleural effusion had regressed (Fig. 1B). CA-125 decreased from 201 UI/L (before starting pembrolizumab) to 169 UI/L (Fig. 1C). A pelvic MRI in July 2018 confirmed the suspect aspect of the left ovarian mass. Immunotherapy was pursued and a laparoscopy with bilateral salpingo-oophorectomy and peritoneal biopsies was performed in September 2018. Pathological analysis revealed the presence of HGSOC in the left ovary, whereas multiple peritoneal biopsies and the right ovary were negative. Cancer cells had solid or glandular proliferation without areas of necrosis, were positive for PAX-8, estrogen receptor and focally positive for WT-1. Progesterone receptor and p53 were negative. Peritoneal cytology was positive for malignant cells.

The initial pleural biopsy was reviewed and compared with the ovarian tumor. It showed that the pleural lesion had comparable morphology and IHC features and was most likely a metastasis of HGSOC (Fig. 1D and 1G). IHC staining of PD-L1 were similar in the ovarian tumor and pleural metastasis (Fig. 1F and 2I). There was massive intraepithelial infiltration by CD8^+^ T cells in both biopsies (≥20 CD8^+^ T cells per field as defined by the Ovarian Tumor Tissue Analysis Consortium) (Ovarian Tumor Tissue Analysis et al., 2017) (Fig. 1E and 1H). However, it seems that upon treatment with pembrolizumab, infiltration by CD8^+^ T cells was reduced while PD-L1 expression did not change.

A comparison of the abdominal CT-scan before (April 2018) and after pembrolizumab (June 2018) showed that the ovarian mass had regressed under immunotherapy while a cystic component developed. After surgery, CA-125 dropped to 53 UI/l (Fig. 1C). Given the effectiveness, the good tolerance to pembrolizumab and the potential toxicity of standard chemotherapy in the context of renal failure, immunotherapy was continued. After 30 cycles of pembrolizumab 200 mg IV every 3 weeks, the patient remained asymptomatic. The disease is still in partial response after 24 months of follow-up (March 2020) with persistence of pleural nodules, stable in size.

Germline sequencing of *BRCA1* and *BRCA2* genes revealed a *BRCA2* variant of unknown significance (p.Thr1011Arg). Molecular analyses of the tumor (NGS400) estimated tumor mutational burden to 2.41 Mbp and identified pathogenic mutations of *TP53* (p.Val143Glyfs) and *NOTCH2* (p.Pro6Argfs) and a missense mutation of *CSMD3* (p.Pro670His). Copy number analysis by SNP array showed numerous copy number alterations without features suggestive of homologous recombination deficiency (Popova et al., 2012). No focal alterations (bi-allelic losses or amplifications) were identified. We did not observe an amplification of the PD-L1 locus (*CD274*). The tumor was tested for microsatellite instability by IHC and the expression of DNA repair proteins was intact.

*CSMD3* is the second most frequently mutated gene in HGSOC according to the TCGA and most mutations are missense (Cancer Genome Atlas Research, 2011), like for our patient. Its mutation is associated with better survival (La Fleur et al., 2019, Deng et al., 2017) and higher response rate to anti-PD1 in solid tumors (Yang et al., 2020). Thus, we looked at transcriptional dysregulations in *CSMD3* mutated HGSOC in the TCGA cohort (*in silico* analysis through www.cbioportal.org) (Cerami et al., 2012). We found that *CCL5, CXCL9* and *GRZMK,* 3 genes linked to infiltration by CD8^+^ T cells and response to immune checkpoint blockade in solid tumors (Dangaj et al., 2019, Gide et al., 2019), were among the top 30 up-regulated genes in *CSMD3* mutated cases (Fig. 1J-L).

## 3 Discussion

In the last decade, immune checkpoint inhibitors targeting PD1 or its ligand PD-L1 have led to dramatic improvement of survival in several metastatic cancers such as renal cell carcinoma and melanoma. Although ovarian cancer is immunogenic (Ovarian Tumor Tissue Analysis et al., 2017, Labidi-Galy et al., 2012, Zhang et al., 2003), the ORR to anti-PD1/PD-L1 in relapsing HGSOC patients does not exceed 10% and it is not a standard of care in this disease. So far, the largest study investigating pembrolizumab as monotherapy in EOC (the KEYNOTE-100 study) enrolled 376 pretreated patients and showed an ORR of 8% (irrespective of platinum-sensitivity). The KEYNOTE-100 study suggested a correlation between ORR and PD-L1 expression: ORR was 17% in patients harboring tumors with a Combined Positive Score (CPS) > 10, compared with 5% if CPS < 1, respectively (Matulonis et al., 2019). Another immune checkpoint inhibitor, anti-PD-L1 avelumab, showed an ORR of 3.4% as monotherapy in women with platinum-resistant or refractory EOC according to the randomized phase III trial JAVELIN Ovarian 200 (Pujade-Lauraine et al., 2019). Although the methods estimating PD-L1 expression vary between the KEYNOTE-100 and JAVELIN Ovarian 200 studies, it appears that EOC that express PD-L1 are more likely to benefit from immunotherapy.

One previous case of a heavily pretreated patient with metastatic HGSOC showed complete response to pembrolizumab. IHC analyses showed high PD-L1 expression in ≥50% of tumor cells and massive intra-epithelial infiltration by T cells (CD4^+^ and CD8^+^) (Bellone et al., 2018). Genomic analysis revealed *CD274* (PD-L1 gene) structural variation causing aberrant overexpression of PD-L1. Our patient had a TPS of 10% but did not carry a *CD274* (PD-L1) gene amplification. However, PD-L1 expression was accompanied by massive intra-epithelial CD8^+^ T cells infiltration in pleural metastases. CD8^+^ T cells are key elements in the control of EOC by the immune system and their presence is associated with better outcomes (Ovarian Tumor Tissue Analysis et al., 2017). The tumor microenvironment of our case appeared to be “T cell inflamed” or “hot tumor”, a key feature for response to anti-PD1 therapy (Zappasodi et al., 2018). We found a missense mutation of *CSMD3*, the second most frequently mutated gene in HGSOC (Cancer Genome Atlas Research, 2011). This gene is mainly expressed in the brain and is located on 8q22.3–q24.1 to which benign adult familial myoclonic epilepsy type I has been mapped (Shimizu et al., 2003). It contains very large introns and its function remains to be determined in cancer. Few data are available on *CSMD3* mutation and tumor microenvironment, although it is among the top mutated genes in cancer. Since its mutation is associated with better survival (Deng et al., 2017; La Fleur et al., 2019) and higher response rate to anti-PD1/PD-L1 in solid tumors (Yang et al., 2020), we questioned whether there could be a link between *CSMD3* mutation and the “hot tumor” features of our case. Interestingly, we found *CCL5* and *CXCL9* among top up-regulated genes in the *CSMD3* mutated cases of the TCGA HGSOC cohort. Up-regulation of these two chemokines is associated with infiltration by CD8^+^ T cells across multiple solid tumors (Dangaj et al., 2019). *CCL5*^*hi*^ *CXCL9*^*hi*^ tumors are immunoreactive and respond to checkpoint blockade (Dangaj et al., 2019). Another up-regulated gene in *CSMD3* mutated HGSOC was *GZMK*, the gene of granzyme K, recently associated with increased response to checkpoint blockade (Gide et al., 2019). Of course our observations are correlative and we did not investigate the levels of expression of *CLL5*; *CXCL9* or *GZMK* in our case. Nevertheless, they bring a plausible explanation of such immunoreactive phenotype and warrant further investigation in larger series of HGSOC treated by anti-PD1/PD-L1.

Besides response to neoadjuvant immunotherapy with pembrolizumab, our case is extraordinary because it was misdiagnosed as a metastatic lung adenocarcinoma. This was due to renal failure that prevented the injection of CT-scan contrast medium and discovery of the ovarian tumor. Since pembrolizumab is approved for metastatic lung adenocarcinoma, she received it without prior chemotherapy.

In conclusion, this is the first case of metastatic *CSMD3* mutated HGSOC with sustained response to anti-PD1 without prior chemotherapy. This case suggests that massive infiltration by CD8^+^ T cells and expression of PD-L1 could predict benefit from neoadjuvant immunotherapy in case of HGSOC. The good tolerance and quality of life are two important factors that favor immunotherapy over chemotherapy, especially in elderly and frail patients who cannot receive standard chemotherapy (3-weeks carboplatin/paclitaxel). Further investigation is warranted to identify patients who would benefit from neoadjuvant immunotherapy in HGSOC and whether *CSMD3* mutated cases are “hot” tumors.

## CRediT authorship contribution statement

**Julie Terzic:** Data curation and Formal analysis, Writing - original draft, Writing - review & editing. **Amanda Seipel:** Data curation and Formal analysis, Funding acquisition, Writing - original draft, Writing - review & editing. **Jean Dubuisson:** Writing - review & editing. **Jean-Christophe Tille:** Data curation and Formal analysis, Funding acquisition, Writing - review & editing. **Petros Tsantoulis:** Writing - review & editing. **Alfredo Addeo:** Writing - review & editing. **S. Intidhar Labidi-Galy:** Conceptualization, Data curation, Formal analysis, Investigation, Methodology, Project administration, Resources, Software, Supervision, Validation, Visualization, Writing - original draft, Writing - review & editing.

## Declaration of Competing Interest

Consulting fees: SILG (MSD and AstraZeneca); PT (BMS, Merck and Roche); AA (BMS, AstraZeneca, MSD, Takeda, Pfizer, Roche and Boehringer Ingelheim).
